# The Effect of Sulfated (1→3)-α-L-Fucan from the Brown Alga *Saccharina cichorioides* Miyabe on Resveratrol-Induced Apoptosis in Colon Carcinoma Cells

**DOI:** 10.3390/md11010194

**Published:** 2013-01-21

**Authors:** Olesia S. Vishchuk, Svetlana P. Ermakova, Tatyana N. Zvyagintseva

**Affiliations:** Laboratory of Enzyme Chemistry, G.B. Elyakov Pacific Institute of Bioorganic Chemistry, Far Eastern Branch, Russian Academy of Sciences, 159 100-Let Vladivostoku Ave., Vladivostok 690022, Russian Federation; E-Mails: swetlana_e@mail.ru (S.P.E.); zvyag@piboc.dvo.ru (T.N.Z.)

**Keywords:** brown alga, *Saccharina cichorioides* Miyabe, fucoidan, resveratrol, colon cancer

## Abstract

Accumulating data clearly indicate that the induction of apoptosis by nontoxic natural compounds is a potent defense against the development and progression of many malignancies, including colon cancer. Resveratrol and the fucoidans have been shown to possess potent anti-tumor activity *in vitro* and *in vivo*. The aim of the present study was to examine whether the combination of a fucoidan from the brown alga *Saccharina cichorioides* Miyabe and resveratrol would be an effective preventive and/or therapeutic strategy against colon cancer. Based on NMR spectroscopy and MALDI-TOF analysis, the fucoidan isolated and purified from *Saccharina cichorioides* Miyabe was (1→3)-α-L-fucan with sulfate groups at C2 and C4 of the α-L-fucopyranose residues. The fucoidan enhanced the antiproliferative activity of resveratrol at nontoxic doses and facilitated resveratrol-induced apoptosis in the HCT 116 human colon cancer cell line. Apoptosis was realized by the activation of initiator caspase-9 and effector caspase-7 and -3, followed by the cleavage of PARP. Furthermore, significant inhibition of HCT 116 colony formation was associated with the sensitization of cells to resveratrol by the fucoidan. Taken together, these results demonstrate that the combination of the algal fucoidan with resveratrol may provide a potential therapy against human colon cancer.

## 1. Introduction

Worldwide, colorectal cancer is one of the most common cancers, with a high propensity to metastasize [1]. Although early-stage colorectal cancer can be successfully treated surgically, advanced-stage colorectal cancer frequently recurs and becomes fatal [2]. For this reason, new therapeutic strategies are needed for the treatment of advanced or metastatic colorectal cancer.

Cancer is a disease state caused by the disruption of cellular homeostasis between cell death and cell proliferation [3]. Apoptosis, a major process of programmed cell death, plays an important role in the regulation of tissue development and homeostasis [4,5], making the induction of apoptosis a useful approach in cancer therapies. The use of synthetic or natural agents, such as cisplatin, etoposide, camptothecin, mitomycin, resveratrol and polyphenols from green tea (EGCG and its derivatives) in cancer therapy is limited by several factors, including toxicity, side effects and drug resistance [6,7]. The search for and isolation of new nontoxic compounds that sensitize cancer cells to apoptosis induction by chemotherapeutic agents are tasks of high importance in the modern strategy of cancer therapy.

Resveratrol is a natural polyphenol that is found in foods and beverages such as grapes, berries, peanuts and wine [8]. Numerous animal and human studies have investigated the effects of resveratrol, the most significant of which include its antioxidant, anti-tumor, cardiovascular and anti-inflammatory activities [9]. The anti-tumor activity of resveratrol has been observed in several human cancer cell lines, including human leukemia [10], breast cancer [11] and colon cancer [12]. The antiproliferative properties of resveratrol are thought to be based on cell cycle regulation and apoptosis induction. However, its use has been limited by the frequent development of drug resistance and toxicity. Chemosensitization, the use of one drug or agent to render cancer cells more susceptible to a second agent, represents a novel strategy to enhance the effects of cancer therapeutics [13].

Fucoidans, sulfated polysaccharides from brown algae, have recently attracted a lot of attention as a nontoxic compound possessing high anti-tumor, immunomodulating, antioxidant and anticoagulant activities [14]. In particular, the anti-tumor activity has attracted considerable attention. Several investigations have found that the fucoidans have antiproliferative activity *in vitro*, as well as inhibitory activity against tumors growing *in vivo* [15] Moreover, these polymers induced apoptosis in several cancer cell lines [16,17]. They exhibit antimetastatic activity by blocking interactions between cancer cells and the basement membrane [18]. Finally, some sulfated algal polysaccharides were found to inhibit angiogenesis by interfering with the binding of vascular endothelial growth factor (VEGF) and basic fibroblast growth factor (bFGF) to their respective receptors [19]. Nevertheless, the question of whether fucoidans are able to enhance the anti-tumor activity of chemotherapeutic agents has not been answered.

In this study, we hypothesize that the fucoidan from the brown alga *Saccharina cichorioides* Miyabe (*Saccharina cichorioides*) will enhance the anti-tumor effect of resveratrol by inducing apoptosis in colon cancer cells. The combination of the fucoidan and resveratrol may be a novel, effective preventive/therapeutic strategy against colon cancer.

## 2. Results and Discussion

### 2.1. Isolation and Characterization of the Fucoidan from *S. cichorioides*

*Saccharina cichorioides* (formerly named *Laminaria cichorioides*), a widely distributed specie of brown alga (order Laminariales) of the Russian Far East, is a rich and easily regenerated source of polysaccharides with unique structures and valuable biological activities.

**Figure 1:**
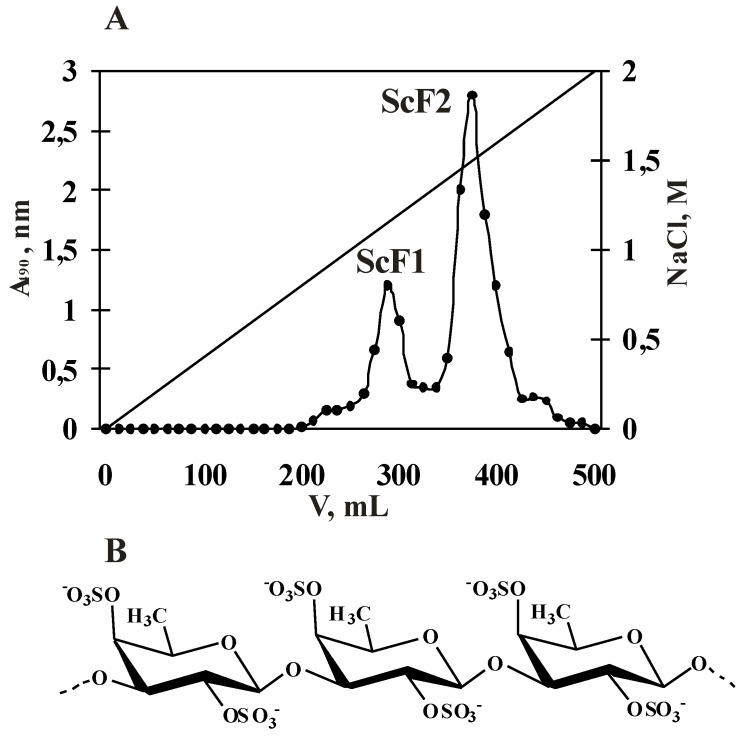
(**A**) Elution profile of the water-soluble polysaccharide fraction after anion-exchange chromatography (DEAE-cellulose) and (**B**) a fragment of the fucoidan structure from the brown alga *Saccharina cichorioides*.

The isolation, purification and determination of the fucoidan structure from *Saccharina cichorioides* were described in our previous studies [20,21]. The fractions ScF1 and ScF2 were obtained after anion-exchange chromatography on DEAE-cellulose and were characterized as sulfated mannofucan and highly sulfated α-L-fucan, respectively (Figure 1A, Table 1).

**Table 1:** Yields and characteristics of polysaccharides fractions from brown alga *Saccharina cichorioides* after anion-exchange chromatography on DEAE-cellulose.

Fraction	Yield *, %	Mw, kDa	Content **, %		Monosaccharide composition, mol%	
			Carbohydrate	SO_3_Na^−^	Fuc	Man
ScF1	1.0	n.d.	55.0	21.0	95.0	5.0
ScF2	2.2	540	32.0	39.0	100.0	0

ScF1—fraction of polysaccharide, eluted with 1.0 M NaCl; ScF2—fraction of polysaccharide, eluted with 1.5 M NaCl; Monosaccharide composition: Fuc = fucose, Man = mannose, SO_3_Na^−^ = sulfate group; *, % of dried defatted algae weight; **, % of sample weight; n.d., not determined.

To investigate biological activity of polysaccharides and establish their structure-activity relationship, it is necessary to isolate homopolysaccharide with a high content of sulfate groups. The fraction of polysaccharide ScF1, obtained after anion-exchange chromatography on DEAE-cellulose, represented sulfated heteropolysaccharide, consisted of fucose, mannose and sulfate groups (Table 1). Whereas the fraction ScF2, also obtained after anion-exchange chromatography, contained only α-L-Fuc*p* residues and sulfate groups (Table 1). That is why fucoidan ScF2 was chosen for further structural characterization.

According to IR spectroscopy, 1D and 2D NMR spectroscopy and MALDI-TOF analysis (data not shown), the fucoidan contained in fraction ScF2 from *Saccharina cichorioides* is a linear (1→3)-linked α-L-fucan in which α-L-fucopyranose (α-L-Fuc*p*) residues are 2,4-disulfated (Figure 1B).

This observation is in agreement with the fact that the above-mentioned structure of the core of the fucoidan is prevalent in the order Laminariales (Phaeophyceae) [22]. One exception is the fucoidans from *Saccharina gurjanovae* f. *lanciformis* (Petrov), *S. japonica* Areschoug and *Undaria pinnatifida* Harvey Suringar (order Laminariales), which are galactofucans [23,24].

The anti-tumor activity of fucoidans has been reported to be closely related to their sulfate content, molecular weight, monosaccharide composition and structure of the main polymer chain [25]. Based on these previous results, the highly sulfated liner polymer from fraction ScF2 was selected for further investigation of its ability to enhance the anti-tumor effect of resveratrol.

### 2.2. Anti-Tumor Activity

#### 2.2.1. Cytotoxicity of the Fucoidan from the Brown Alga *Saccharina cichorioides* and Resveratrol

The primary aim of this investigation was to determine whether a combination of the fucoidan from *Saccharina cichorioides* and resveratrol would be a better outcome for colon cancer prevention. As a first step toward accomplishing this aim, we examined the cytotoxicity of each compound alone against human colon carcinoma cell line HCT 116 cells. This cell line is known to be invasive and highly motile *in vitro* studies.

To define the inhibitory concentration (IC_50_) of fucoidan ScF2 and resveratrol, HCT 116 cells were treated with ScF2 (100, 200, 400, 800 μg/mL) and resveratrol (5, 10, 20, 40, 80 μM) for 24 h as described in the Experimental section.

**Figure 2:**
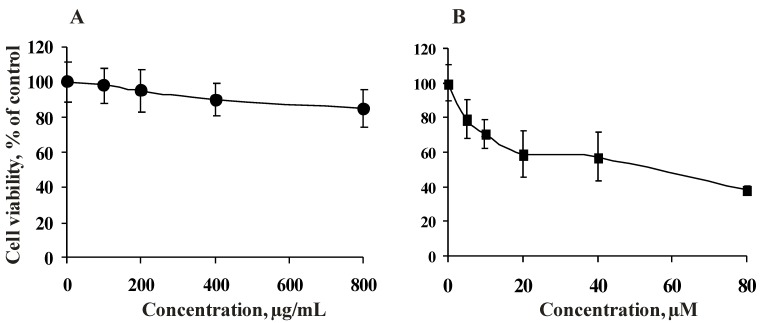
Cytotoxicity of the fucoidan from the brown alga *Saccharina cichorioides* and resveratrol. HCT 116 cells (1.0×10^4^) were incubated with (**A**) fucoidan ScF2 (100–800 μg/mL) and (**B**) resveratrol (5–80 μM) for 24 h at 37°C in a 5% CO_2_ incubator. Compound cytotoxicity was estimated using the MTS assay. Data are represented as the mean ± SD as determined from triplicate experiments.

Fucoidan ScF2 (800 μg/mL) was less cytotoxic against HCT 116 cells (the cell number was reduced 15%) (Figure 2A), while resveratrol (IC_50_ value of 55 μM) possessed cytotoxic activity against the tested cell line, as indicated by the dose-response curve (Figure 2B).

These results are in agreement with data from previous studies. The sulfated polysaccharides from other species of brown algae were found to be nontoxic against JB6 Cl41 (epidermal mouse cells), Vero (African green monkey kidney), MCF-10A (human epithelial cells), MCF-7 (human breast cancer cells) and other cells [26,27]. It has been reported that resveratrol was cytotoxic against different types of cancer; for example, the IC_50_ value of resveratrol against HCT 116 cells ranged from 35 to 100 μM [28,29].

In the present study, we observed that fucoidan ScF2 is nontoxic against HCT 116 cells in doses up to 800 μg/mL, which indicated that the anti-tumor activity of this polysaccharide may be produced by activation of specific receptors and proteins, suppression of signal transduction and/or induction of apoptosis in cancer cells. These findings make the fucoidan an ideal candidate for further studies as an anti-tumor agent, which could enhance the anti-tumor activity of resveratrol at nontoxic doses.

Based on the cytotoxic assay data, a fucoidan concentration of 300 μg/mL (the cell number was reduced less than 10%) and a resveratrol concentration of 40 μM (the cell number was reduced less than 50%) were chosen for the antiproliferative assay.

#### 2.2.2. Antiproliferative Activity of the Fucoidan from the Brown Alga *Saccharina cichorioides* and Resveratrol

**Figure 3:**
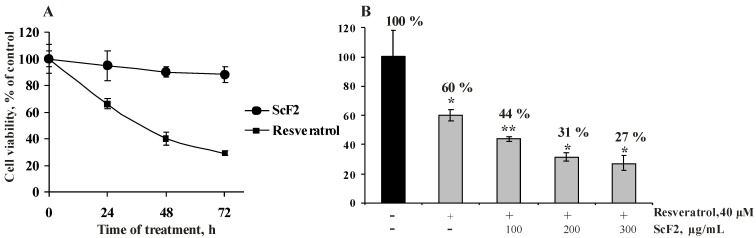
Effect of fucoidan ScF2, resveratrol and a combination of resveratrol and fucoidan ScF2 on cell proliferation. (**A**) HCT 116 cells (8.0×10^3^) were treated with fucoidan ScF2 (300 μg/mL) and resveratrol (40 μM) for 24, 48 and 72 h. (**B**) HCT 116 cells were pre-treated with resveratrol (40 μM) for 24 h and then incubated with fucoidan ScF2 (100, 200 and 300 μg/mL) for an additional 24 h. Cell viability was estimated using the MTS assay. Data are represented as the mean ± SD as determined from triplicate experiments. A Student’s *t*-test was used to evaluate the data with the following significance levels: *p < 0.05, **p < 0.01, ***p < 0.001.

The effects of each compound alone on the growth of HCT 116 cells were studied. The cells were treated with fucoidan ScF2 (300 μg/mL) and resveratrol (40 μM) for 24, 48 and 72 h (Figure 3A).

The incubation of HCT 116 cells with fucoidan ScF2 affected cell growth slightly (12% growth inhibition after 72 h of treatment). Resveratrol inhibited the proliferation of HCT 116 cells by 34%, 60% and 71% after 24, 48 and 72 h, respectively (Figure 3A).

Based on these results, the optimal treatment time and concentrations of fucoidan ScF2 and resveratrol were chosen to determine whether the fucoidan from *Saccharina cichorioides* might enhance the antiproliferative effect of resveratrol. Cells were pre-incubated with resveratrol (40 μM) for 24 h and then treated with fucoidan ScF2 (100, 200 and 300 μg/mL) for an additional 24 h. As shown in Figure 3B, the growth inhibitory effect of the combination of fucoidan ScF2 and resveratrol was greater than the effect of either agent alone. The antiproliferative activity of resveratrol increased by 13%, 18% and 22% after the addition of 100, 200 and 300 μg/mL fucoidan ScF2, respectively (Figure 3B). So, it became evident that the inhibition of cell proliferation was stronger as the concentration of fucoidan ScF2 increased.

The growth inhibitory activities of resveratrol and fucoidans from different species of brown algae have been reported in recent years. These natural compounds have been shown to suppress viability in a wide variety of cancer cells, including lymphoid and myeloid, breast, colon, pancreas, stomach, prostate, lung and cervical cancers [8,30].

However, this study is the first to demonstrate the ability of the fucoidan from the brown alga *Saccharina cichorioides* to enhance the antiproliferative activity of resveratrol at nontoxic doses.

#### 2.2.3. The Fucoidan from the Brown Alga *Saccharina cichorioides* Facilitates Resveratrol-Induced Apoptosis in Colon Cancer Cells

The next experiment was performed to determine whether the comparatively greater growth inhibition of colon cancer cells in response to the combinatorial treatment with fucoidan ScF2 and resveratrol could be the result of increased apoptosis.

Apoptosis is a representative form of programmed cell death, which is thought to be critical for cancer prevention [31]. Current studies indicate that there are two main apoptotic pathways: the extrinsic or death receptor pathway and the intrinsic or mitochondrial pathway. These two apoptotic pathways converge on caspase-3 and other subsequent proteases and nucleases that drive the terminal events of apoptosis. Caspases perform critically important roles in the induction of apoptosis. They are classified based on their mode of activation as either initiator (caspase-8 and caspase-9) or effector caspases (caspase-3 and caspase-7). Activated initiator caspases can cleave and activate effector caspases, which in turn cleave a variety of cellular substrates, most notably poly(ADP-ribose) polymerase (PARP). One of the most important functions of PARP is to help repair single-strand DNA nicks; thus, cleaved PARP is a useful marker for apoptosis [32].

Based on these previous results, we attempted to determine whether fucoidan ScF2, resveratrol or a combination of these two compounds activated initiator and effector caspases as well as PARP cleavage via Western blotting and an immunoprecipitation assay.

**Figure 4:**
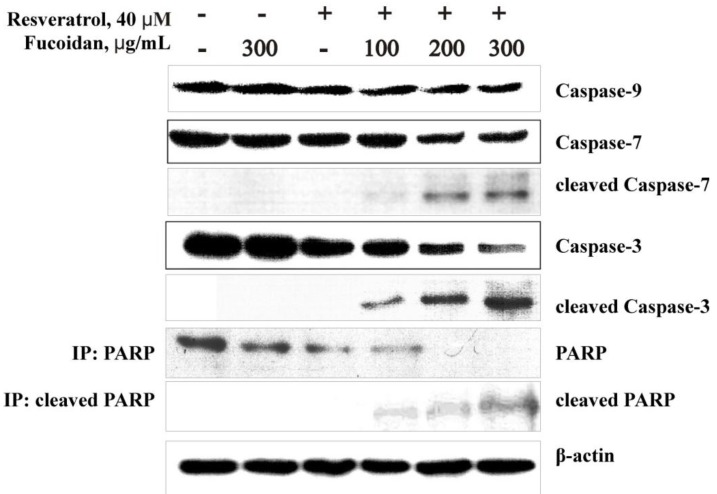
Effect of the fucoidan on resveratrol-induced apoptosis in colon cancer cells. HCT 116 cells were either treated or not treated with 40 μM resveratrol for 24 h and then treated or not treated with 100, 200, 300 μg/mL of fucoidan ScF2 for an additional 24 h. After treatment, total protein lysates were prepared. The protein samples (30 μg) were subjected to SDS-PAGE and followed by detection by immunoblotting using antibodies against caspase-9 (47 kDa), -7 (35 kDa), -3 (35 kDa), cleaved caspase-9 (37, 17 kDa), -7 (20 kDa), -3 (19 kDa), PARP (116 kDa) and cleaved PARP (89 kDa).

The results indicated that resveratrol (40 μM) could induce slight activation of caspase-9, -7 and -3 in HCT 116 cells, and fucoidan ScF2 (300 μg/mL) had no effect on the activation of these caspases (Figure 4). However, the combination of fucoidan ScF2 and resveratrol induced a dose-dependent increase in the activation of caspase-9, -7 and -3. Moreover, the combination of fucoidan ScF2 and resveratrol induced a dose-dependent increase in the protein levels of cleaved caspases-9, -7 and -3 and cleaved PARP in HCT 116 cells (Figure 4). These results suggested that the fucoidan from the brown alga *Saccharina cichorioides* could enhance resveratrol-induced apoptosis in human colon cancer cells by activating initiator and effector caspases and cleaving PARP, which ultimately cause the morphological and biochemical changes observed in apoptotic cells.

**Figure 5:**
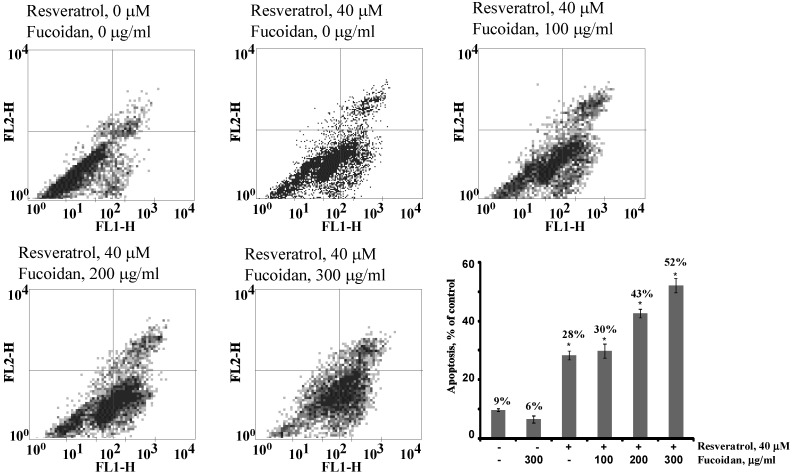
Fluorescence-activated cell sorting (FACS) analysis. FACS analysis of apoptotic cells after addition of the fucoidan (100, 200, 300 μg/mL) in the presence or absence of resveratrol (40 μM). After treatment, HCT 116 cells were labeled with annexin V-FITC and propidium iodide. The distribution pattern of live and apoptotic cells was determined by FACS analysis. In the bottom left quadrants, viable cells display low annexin or no annexin and propidium iodide staining; in the bottom right quadrants, early-stage apoptotic cells display high annexin and low propidium iodide staining; in the top right quadrants, late-stage apoptotic cells display high annexin and high propidium iodide staining; in the top left quadrants, necrosis is represented by high propidium iodide and low annexin staining. The percentage of apoptosis in treated cells compared with untreated cells is representative of at least three independent experiments.

To further test this idea, we investigated the effect of fucoidan ScF2 on resveratrol-induced apoptosis by flow cytometry analysis. As illustrated in Figure 5, the treatment with resveratrol alone yielded 28% apoptotic cells. On the other hand, the treatment of resveratrol-treated cells with fucoidan ScF2 at 100, 200 and 300 μg/mL increased the percentage of apoptotic cells to 30%, 43% and 52%, respectively (Figure 5).

These results strongly indicated that fucoidan ScF2 facilitated resveratrol-induced apoptosis in the HCT 116 cell line and that this strategy might be a novel one for improving the treatment of colon cancer.

Because apoptosis induction by nontoxic natural agents was found to be arguably the most potent defense against cancer, many studies have focused on elucidating the proapopotic activity of many biologically active compounds, such as the fucoidans from brown algae and resveratrol. The molecular mechanism of resveratrol-induced apoptosis was studied in detail. The growth-inhibitory effects of resveratrol were thought to be mediated by cell-cycle arrest, the upregulation of p21Cip1/WAF1 and proteins p53 and Bax, the downregulation of survivin, cyclin D1, cyclin E, Bcl-2, Bcl-xL and cIAPs, and the activation of caspases [33].

Previous studies have shown that the fucoidans induced apoptosis in different types of cancer cells via a variety of mechanisms. It was reported that commercially available (Sigma, USA) fucoidan (100 μg/mL) from the brown alga *Fucus vesiculosus* Linnaeus induced apoptosis via the activation of caspase-3 and downregulation of the ERK pathway in human lymphoma HS-Sultan cells [34] and via the caspase-8-dependent pathway in MCF-7 breast cancer cells [35]. Commercially available fucoidan (820 μg/mL), sold under the product name “Power the fucoidan” from *Cladosiphon novae*-*caledoniae* Kylin (Daiichi Sangyo Corporation, Japan) activated a caspase-independent apoptotic pathway in MCF-7 breast cancer cells via the activation of ROS-mediated MAP kinases and the regulation of the Bcl-2 family protein-mediated mitochondrial pathway [17]. Galactofucan (200 μg/mL) from *Undaria pinnatifida* was found to induce apoptosis in A549 human lung carcinoma cells through the downregulation of Bcl-2 and the activation of the caspase pathway [36]. Several experiments were performed to investigate the anti-tumor effect of the fucoidan from *F. vesiculosus* on colon cancer. Hyun *et al.* [37] reported that the fucoidan (100 μg/mL) induced apoptosis in HCT-15 human colon carcinoma cells via the activation of caspase-9 and -3 accompanied by changes in Bcl-2 and Bax; additionally, there were changes in the phosphorylation of ERK, p38 kinase and Akt. Another study demonstrated that this fucoidan (20 μg/mL) induced apoptosis in HT-29 human colon carcinoma cells and HCT 116 cells via both the death receptor-mediated apoptotic pathway and mitochondria-mediated apoptotic pathway [16].

However, it should be noted that most of these studies have used a commercially available fucoidan from *Fucus vesiculosus*, which contains more than 16 different fucans with varying proportions of the individual monosaccharide [38]. The anti-tumor activity of the fucoidans, including apoptosis induction, is known to depend on their structural characteristics. These characteristics could vary according to the algae species, season of harvest, age of the plants and even parts of a single algal thallus [39]. Each newly isolated fucoidan is therefore a new compound with unique structural characteristics, thus representing a potential novel drug. To the best of our knowledge, this work is the first to show that purified fucoidan from the brown alga *Saccharina cichorioides* significantly facilitated resveratrol-induced apoptosis in human colon cancer cells via the caspase-3 activation-dependent pathway.

#### 2.2.4. The Fucoidan from the Brown Alga *Saccharina cichorioides* Sensitizes Human Colon Cancer Cells to Resveratrol

The search for compounds that sensitize cancer cells to chemotherapeutic agents is an important task in the modern strategy of anticancer therapy. Recently, it has been reported that sulfated polysaccharides from brown algae inhibited colony formation of human breast cancer and melanoma cells [40,41]. Resveratrol is able to suppress cell transformation induced by different stimulants [42,43]. Based on these studies, we suggest that a combination therapy that includes the fucoidan from the brown alga *Saccharina cichorioides* and resveratrol will be highly effective in inhibiting colony formation of colon cancer cells.

**Figure 6:**
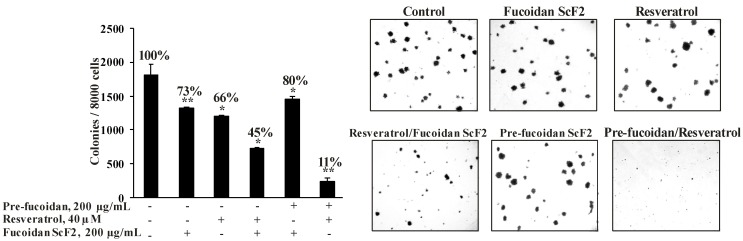
The effect of fucoidan ScF2, resveratrol, and the combination of resveratrol and fucoidan ScF2 on colony formation of human colon cancer cells. HCT 116 human colon cancer cells (2.4×10^4^/mL) were either treated or not treated with fucoidan ScF2 (200 μg/mL), resveratrol (40 μM) and resveratrol with fucoidan ScF2 together in 1 mL of 0.3% Basal Medium Eagle (BME) agar containing 10% FBS, 2 mM L-glutamine and 25 μg/mL gentamicin. The cultures were maintained at 37°C in a 5% CO_2_ incubator for 14 days. The cell colonies were scored using a Motic AE 20 microscope (China) and the Motic Image Plus computer program. All assays were performed at least in triplicate. The results are expressed as the mean ± standard deviation (SD). A Student’s *t*-test was used to evaluate the data with the following significance levels: *p < 0.05, **p < 0.01, ***p < 0.001.

To evaluate the inhibitory effects of fucoidan ScF2, resveratrol, and fucoidan ScF2 and resveratrol together on colony formation, we performed soft agar clonogenic assays using HCT 116 cells (Figure 6). In the preliminary study, we found that fucoidan ScF2 concentration effective against colony formation was 200 μg/mL; this concentration was therefore used in additional experiments. Resveratrol was used at a concentration of 40 μM as in the antiproliferative assay.

The treatment of HCT 116 cells with resveratrol (40 μM) and fucoidan ScF2 (200 μg/mL) alone reduced the colony number by 34% and 27%, respectively, whereas the combination of these two agents reduced the colony number by 60% (Figure 6).

In this study, we also attempted to investigate whether the fucoidan could sensitize colon cancer cells to resveratrol. It was found that pre-incubation of HCT 116 cells with fucoidan ScF2 (200 μg/mL) for 24 h significantly sensitized colon cancer cells to resveratrol. This pre-incubation reduced the colony number by more than 80% and reduced colony size when compared to cells treated with either resveratrol or fucoidan ScF2 alone and even treatment with a combination of the fucoidan and resveratrol at the same time (Figure 6). The present findings demonstrate for the first time that human colon cancer cells treated with the fucoidan and resveratrol underwent a significant reduction in their ability to express their transformed phenotype.

Information about the ability of the fucoidans to sensitize cancer cells to chemotherapeutic drugs and/or enhance their anti-tumor activity is very limited. Philchenkov *et al.* [44] used human malignant MT-4 lymphoid cells to study the ability of the fucoidan from *Fucus evanescens* C.Agardh to modulate the apoptosis induced by etoposide, which is a potent chemotherapeutic agent with clinical activity against a broad range of human malignancies. It was shown that the incubation of MT-4 lymphoid cells with the fucoidan prior to etoposide treatment increased the apoptosis percentage and activated caspase-3, which was not observed with etoposide treatment only. However, the fucoidan’s ability was assessed in cells pretreated for 14 days with a high concentration of the fucoidan (500 μg/mL). Therefore, our findings that a low concentration of the highly sulfated α-L-fucan from the brown alga *Saccharina cichorioides* significantly sensitized the HCT 116 cells to resveratrol may lead to it as a novel therapy for colon cancers in the future.

## 3. Experimental Section

### 3.1. Chemicals and Cell Culture Reagents

DEAE-cellulose and 10 kDa membrane were purchased from Sigma (St. Louis MO, USA) and Supelco (Bellefonte, PA, USA), respectively. Organic solvents, inorganic salts and acids were manufactured by Diaem (Moscow, Russian Federation).

The MTS reagent 3-(4,5-dimethylthiazol-2-yl)-5-(3-carboxymethoxyphenyl)-2-(4-sulfophenyl)-2*H*-tetrazolium, inner salt and resveratrol were purchased from Promega (Madison, WI, USA) and Sigma (St. Louis, MO, USA), respectively.

Antibodies against caspase-9, caspase-7, caspase-3, cleaved caspase-9, cleaved caspase-7, cleaved caspase-3, poly(ADP-ribose) polymerase (PARP), cleaved PARP, β-actin and horseradish peroxidase (HRP)-conjugated secondary antibody from rabbit and mouse were obtained from Cell Signaling Technology (Massachusetts, USA).

The ECL Plus chemiluminescence detection kit and Annexin V-FITC apoptosis detection kit were from Amersham (Pittsburgh, PA, USA) and MBL (Watertown, MA, USA), respectively.

Basal Medium Eagle (BME), McCoy’s 5A Modified Medium (McCoy’s 5A), phosphate buffered saline (PBS), L-glutamine, penicillin-streptomycin solution, trypsin, fetal bovine serum (FBS), sodium hydrocarbonate (NaHCO_3_) and agar were purchased from Biolot (Moscow, Russian Federation).

### 3.2. Algal Material

The brown alga *Saccharina cichorioides* Miyabe (Phaeophyta/Laminariales/Laminariaceae) was collected in July of 2005 in Trinity Bay (Sea of Japan) at the Sea Experimental Station of G.B. Elyakov Pacific Institute of Bioorganic Chemistry, Far Eastern Branch of the Russian Academy of Sciences (PIBOC FEB RAS). The fresh algae was freed from attached impurities, washed thoroughly with tap water and air-dried.

### 3.3. Cell Lines

The HCT 116 cells (ATCC^®^No. CCL-247) were obtained from the American Type Culture Collection (ATCC, USA).

### 3.4. Isolation and Determination of the Fucoidan Structure

#### 3.4.1. Extraction of Water-Soluble Polysaccharide from the Brown Alga *S. cichorioides*

Isolation of polysaccharides from the brown alga *Saccharina cichorioides* was carried out using the modified method [45]. Briefly, the alga was ground to a particle size 1 mm and treated with ethanol, acetone and chloroform sequentially. Samples of defatted, dried alga (100 g) were extracted with 0.1 N HCl (2 L) at room temperature. The extracts were concentrated to 1/5 of the volume by ultrafiltration using a 10 kDa Millipore membrane, and the polysaccharides were then precipitated with four volumes of 96% ethanol. The precipitates were washed with 96% ethanol and air-dried.

#### 3.4.2. Anion-Exchange Chromatography

Polysaccharide solutions in 0.04 N HCl (1 g/20 mL) were applied onto a DEAE-cellulose column (Cl^−^ form, 3 × 14 cm) equilibrated with 0.04 N HCl. Sulfated polysaccharides were successively eluted with liner gradient of 250 mL H_2_O/250 mL NaCl (2 M). The fractions were dialyzed and lyophilized to obtain the polysaccharide fractions ScF1 and ScF2.

#### 3.4.3. Carbohydrate Content

The carbohydrate content was determined colorimetrically by the phenol-sulfuric acid method [46].

#### 3.4.4. Sulfate Group Content

The sulfate group content was determined by the turbidimetric method after hydrolysis of the corresponding fractions with HCl (1 N) [47].

#### 3.4.5. Acid Hydrolysis

Total acid hydrolysis of fucoidan ScF2 (5 mg) was carried out using trifluoroacetic acid (2 N, 0.5 mL, 6 h, 100 °C), and the acid was evaporated using a 2.5% ammonia solution on a rotary evaporator.

#### 3.4.6. Analysis of Monosaccharide Composition

The monosaccharide composition of the polysaccharides after acidic hydrolysis was determined on a Biotronik IC-5000 carbohydrate analyzer (Germany) using a Shim-pack ISA-07/S2504 (0.4 × 25 cm) column. Elution was performed with a potassium borate buffer at an elution rate of 0.6 mL/min. Detection was carried out by the bicinchoninate method and integration on a Shimadzu C-R2 AX system. Monosaccharides (Rha, Man, Fuc, Gal, Xyl, and Glc) were used as HPLC standards [48].

#### 3.4.7. Desulfation of the Fucoidan

Fucoidan ScF2 (10 mg/mL) was applied onto a cation-exchange column (Timberlite CG-120, 200–400 mesh, Serva, Germany), and pyridine (0.5 mL) was added after elution of the samples. The samples were lyophilized, and 18 mL of DMSO and 2 mL of methanol were added to the fucoidan. This mixture was heated at 100 °C for 3 h. To remove DMSO at the end of the reaction, the mixture was dialyzed then lyophilized.

#### 3.4.8. Determination of Molecular Weight

The molecular weight of fucoidan ScF2 was determined by HPLC on a Shimadzu LC-20A instrument (Japan) with a RID-10A refractometric detector. Polysaccharides were separated over Shodex Asahipak GS-520 HQ and Shodex Asahipak GS-620 columns (7.5 mm × 300 mm) (Showa, Denko, Japan) at 50 °C and eluted with water (0.8 mL/min). The columns were calibrated using a set of P-82 Shodex Standards Pullulan (Japan) and Blue Dextran (Amersham, Sweden).

#### 3.4.9. IR Spectroscopy

The IR spectrum of the fucoidan was registered in KBr pellets on a Carl Zeiss IR-75 spectrometer.

#### 3.4.10. NMR Spectroscopy

NMR spectra for solutions of the fucoidan in D_2_O were obtained on a Bruker Avance DPX-500 NMR spectrometer with a working frequency of 75.5 MHz at 60 °C.

### 3.5. Biological Assays

#### 3.5.1. Cell Culture

HCT 116 human colon cancer cells were cultured in McCoy’s 5A medium supplemented with 10% fetal bovine serum (FBS) and penicillin-streptomycin solution. The cultures were maintained at 37 °C in a 5% CO_2_ incubator (MCO-18AIC, “Sanyo”, Japan).

Resveratrol was dissolved in DMSO and subsequently diluted with McCoy’s 5A medium (final DMSO concentration in experiments was 0.05%), and fucoidan ScF2 was dissolved in McCoy’s 5A medium.

#### 3.5.2. Cytotoxicity Assay

The MTS assay was used as an indicator of cell viability as determined by the mitochondrial-dependent reduction of formazan [49]. Briefly, HCT 116 cells (1 × 10^4 ^cells/200 μL) were seeded in 96-well plates and cultured for 24 h at 37 °C in a 5% CO_2_ incubator. McCoy’s 5A medium was replaced with 200 μL of fresh medium (control), medium containing fucoidan ScF2 (100, 200, 400, 800 μg/mL) or medium containing resveratrol (5, 10, 20, 40, 80 μM). After 24 h, the MTS reagent (20 μL) was added to each well, and cells were incubated for 3 h at 37 °C in a 5% CO_2 _incubator. The intensity of the developed color, which is a reflection of number of live cells, was measured at a wavelength of 490/630 nm in a microplate reader (Power Wave XS, USA). The 50% inhibitory concentration (IC_50_) was defined as the concentration of the fucoidan or resveratrol that caused a 50% reduction in cell viability. All tested samples were assayed in triplicate.

#### 3.5.3. Growth Inhibition Assay

HCT 116 human colon cancer cells were seeded at a density of 8.0 × 10^3^ cells/200 μL of complete McCoy’s 5A medium in 96-well plates. After 24 h, incubation was continued for 24, 48 or 72 h in the absence (control) or presence of fucoidan ScF2 (300 μg/mL) or resveratrol (40 μM) alone. To estimate whether fucoidan ScF2 might enhance the antiproliferative effect of resveratrol, HCT 116 cells (8.0 × 10^3^ cells/200 μL) were pre-treated with resveratrol (40 μM) for 24 h. Culture medium was then substituted with medium containing fucoidan ScF2 (100, 200 and 300 μg/mL), and the cells were incubated for an additional 24 h. At the end of the incubation, the reaction was terminated by adding MTS reagent (20 μL) to each well. Absorbance was measured at 490/630 nm. All assays were performed in triplicate.

#### 3.5.4. Western Blotting Assay

HCT 116 human colon cancer cells (6.0 × 10^5^/10 mL) were seeded in 10-cm dishes for 24 h. Cultures were then treated with McCoy’s 5A medium (control) or resveratrol (40 μM). After 24 h, the cultures were treated with McCoy’s 5A medium (control) or fucoidan ScF2 (100, 200, 300 μg/mL) for an additional 24 h. The cells were collected and washed with ice-cold phosphate-buffered saline (1× PBS) and lysed with lysis buffer (50 mM Tris-HCl (pH 7.4), 150 mM NaCl, 1 mM EDTA, 1 mM EGTA, 10 mg/mL aprotinin, 10 mg/mL leupeptin, 5 mM phenylmethanesulfonylfluoride (PMSF), 1 mM dithiothreitol (DTT) and 1% Triton X-100). Insoluble debris was removed by centrifugation at 12,000 rpm for 10 min, and protein content was determined using Bradford reagent Bio-Rad (Hercules, CA, USA) [50].

Equal amounts of protein (30 μg) were separated electrophoretically by 10%–15% sodium dodecyl sulfate-polyacrylamide gel electrophoresis (SDS-PAGE) and transferred to polyvinylidene fluoride membranes (PVDF). Membranes were subsequently blocked with 5% skim milk in PBST (1× PBS, 0.05% Tween 20) and hybridized with the primary antibodies against caspase-9 (1:1000), caspase-7 (1:1000), caspase-3 (1:1000), cleaved caspase-9 (1:1000), cleaved caspase-7 (1:1000), cleaved caspase-3 (1:1000) and β-actin (1:500) overnight at 4 °C. After thorough washing with PBST, horseradish peroxidase (HRP)-conjugated secondary antibody from rabbit or mouse (1:5000) was applied, and immune complexes were visualized using the enhanced chemiluminescence (ECL) detection system according to the manufacturer’s instructions Amersham (Buckinghamshire, HP7 9NA, UK).

#### 3.5.5. Immunoprecipitation Assay

HCT 116 cells were treated with resveratrol and/or fucoidan ScF2 as stated above (Western blotting assay) and then lysed with lysis buffer (1× PBS, 0.5% sodium deoxycholate, 0.1% SDS, 1% Triton X-100, 10 mg/mL aprotinin, 10 mg/mL leupeptin and 1.6 mg/mL protease inhibitor cocktail. Cell lysates were incubated with antibodies against PARP and cleaved PARP overnight at 4 °C and then with protein A/G-Sepharose beads (Santa Cruz Biotechnology) for an additional 1 h. After centrifugation, the beads were washed three times with wash buffer and resuspended in SDS sample buffer. After heating at 95 °C for 5 min, the samples were loaded onto a 10% gel, and the Western blotting assay was performed.

#### 3.5.6. Flow Cytometry Assay

Apoptosis was detected using the annexin V-FITC apoptosis detection kit as recommended by the manufacturer MBL International Corp. (USA). Apoptosis was compared in HCT 116 cells that were not treated (control) or treated with fucoidan ScF2 (300 μg/mL), resveratrol (40 μM) or resveratrol (40 μM) and fucoidan ScF2 (100, 200, 300 μg/mL) for 48 h. Cells were harvested with 0.025% trypsin/5 mM EDTA in PBS, and 2.5% FBS in PBS was added as soon as the cells were released from the dish. The cells were then transferred to a centrifuge tube, washed with PBS and incubated for 5 min at room temperature with annexin V-FITC plus propidium iodide according to the manufacturer’s protocol. Cells were analyzed on a Becton Dickinson FACSCalibur flow cytometer (BD Biosciences, USA), placing the FITC signal in FL1 and the propidium iodide signal in FL2. Intact cells were gated in the forward/side scatter plot to exclude small debris.

#### 3.5.7. Soft Agar Clonogenic Assay

HCT 116 human colon cancer cells (2.4 × 10^4^/mL) were seeded in 6-well plates and not treated (control) or treated with fucoidan ScF2 (200 μg/mL), resveratrol (40 μM) or resveratrol and fucoidan ScF2 in 1 mL of 0.3% Basal Medium Eagle (BME) agar containing 10% FBS, 2 mM L-glutamine, and 25 μg/mL gentamicin. The cultures were maintained at 37 °C in a 5% CO_2_ incubator for 14 days and the cell colonies were scored using a Motic AE 20 microscope (China) and the Motic Image Plus computer program.

To estimate the ability of the fucoidan to promote resveratrol-inhibited colony formation, HCT 116 сells (6.0 × 10^5^/10 mL) were plated in 10-cm dishes and cultured in McCoy’s 5A medium with 10% FBS at 37 °C. After incubation for 24 h, the cells were pretreated with fucoidan ScF2 (200 μg/mL) for 24 h. The cells were collected by trypsinization and subjected to the soft agar clonogenic assay described above [51].

### 3.6. Statistical Analysis

All assays were performed at least in triplicate. The results are expressed as the mean ± standard deviation (SD). A Student’s *t*-test was used to evaluate the data with the following significance levels: * *p* < 0.05, ** *p* < 0.01 and *** *p* < 0.001.

## 4. Conclusion

The results of this study demonstrate that the combination therapy of the fucoidan from the brown alga *Saccharina cichorioides* and resveratrol at nontoxic doses was highly effective at inhibiting the growth and colony formation of colon cancer cells, which could be attributed to the sensitization of colon cancer cells to resveratrol-induced apoptosis by the fucoidan. Apoptosis was caused by the activation of initiator caspase-9 and effector caspase-7 and -3, followed by cleavage of PARP. We are optimistic that cotreatment with the fucoidan and resveratrol may potentially prove useful in the prevention of colon cancer. However, further study is needed to determine whether the fucoidan could enhance the anti-tumor activity of resveratrol *in vivo* and in humans.
